# Perceptions of primary care among medical students in Lima, Peru: a cross-sectional study in two universities

**DOI:** 10.1017/S146342362610084X

**Published:** 2026-03-09

**Authors:** Sebastian A. Medina-Ramirez, Camila A. Arones-Santayana, Alvaro Taype-Rondan

**Affiliations:** 1 Center for Research in Primary Health Care (CINAPS), Universidad Peruana Cayetano Heredia, Peru; 2 Grupo de investigación P53, Facultad de Medicina Humana, Universidad Peruana Union, Lima, Peru; 3 Facultad de Medicina Alberto Hurtado, Universidad Peruana Cayetano Heredia, Lima, Peru; 4 Unidad de Investigación para la Generación y Síntesis de Evidencias en Salud, Vicerrectorado de Investigación, San Ignacio de Loyola University: Universidad San Ignacio de Loyola, Peru; 5 EviSalud - Evidencias en Salud, Lima, Peru

**Keywords:** medical education, medical student, perception, primary health care

## Abstract

**Objective:**

To describe the perception of primary care (PC) among medical students from two universities in Peru.

**Methods:**

A cross-sectional study was conducted among third- to seventh-year medical students from two universities in Lima, Peru. A questionnaire was applied to evaluate perceptions of PC. Crude and adjusted prevalence ratios (aPR) with their 95% confidence intervals (95% CI) were calculated to assess factors associated with a favorable perception.

**Results:**

Data from 418 medical students were analyzed (women: 60.8%, mean age: 23.4 years). Only 2.2% expected to work in PC after graduation. Regarding perceptions of PC, 82% agreed or strongly agreed that PC is a preparatory step toward medical residency, 55% felt cases were less interesting, and 44% believed the income was lower compared to hospital work. Being enrolled at Universidad Peruana Unión (aPR: 3.35, 95% CI: 1.85–6.05) and having completed an external rotation in PC (aPR: 1.36, 95% CI: 1.03–1.80) were associated with a favorable perception.

**Conclusion:**

Among the assessed students, most viewed PC as a step toward residency, and nearly half considered cases less interesting and income lower compared to hospital work. A favorable perception was associated with university affiliation and having completed external rotations in PC during training.

## Introduction

Primary care (PC) serves as the main gateway to the health system, with the capacity to resolve up to 85% of the population’s health problems (Rosas Prieto *et al.*, 2013; Kruk *et al.,* 2018). Its role includes integrating health promotion, disease prevention, early diagnosis, and management – particularly among vulnerable populations and those with limited access to specialized services (Vignolo *et al.*, 2011; World Health Organization, n.d.).

Despite its importance, the PC implementation faces multiple structural and professional challenges. Among these is the limited availability of diagnostic and therapeutic technologies, which undermines the resolution capacity of first-level facilities and leads to unnecessary referrals for cases that could be managed locally. In addition, the lack of incentives for medical professionals, both financial and in terms of recognition and career advancement reduces the appeal of working at this level (Starfield *et al.*, 2005; Beaulieu *et al.*, 2008).

This situation is further exacerbated by medical education that is predominantly hospital-centered, limiting students’ exposure to community-based care and reinforcing negative perceptions of primary care. Moreover, the emigration of health professionals in search of better working conditions, professional growth, and financial stability further compounds the issue. As a result, there is a persistent lack of interest among physicians in working in primary care, contributing to workforce shortages and weakening the system’s ability to address population health needs through a preventive and territorial approach (Román A *et al.*, 2007; Puertas and Rivera, 2016; Kruk *et al.*, 2018; World Health Organization, n.d.).

Internationally, perceptions of PC vary considerably. In developed countries with integrated health systems and strong primary care policies, such as Canada, the United Kingdom or the Nordic countries, this area is valued as a strategic specialty, with specialized training, where physicians at this level are recognized among professionals (Starfield *et al.*, 2005). In contrast, in middle- and low-income countries such as Latin America, PC continues to be perceived as a less prestigious option, with limited opportunities for professional development and recognition (Llanos Zavalaga *et al.*, 2020; Espinoza-Portilla *et al.*, 2021).

While some studies have examined medical students’ perceptions of PC, a deeper understanding of the specific factors influencing these perceptions remains necessary. Existing research on this topic is limited and has primarily employed qualitative methodologies. Studies conducted in the United States (Riesenberg *et al.*, 2001; Phillips *et al.*, 2012), Europe (Chung *et al.*, 2016; Reid and Alberti, 2018), and parts of Asia (Ohta *et al.*, 2019; Zainal and Smith, 2020) have explored these perceptions with varying focuses. In Latin America, one study found that fewer than half of the participants held a favorable view of primary care. Country-specific findings showed particularly low percentages in Ecuador (11.9%), followed by Peru (35.5%), and Venezuela and Bolivia (both 37%) (Pereyra-Elías *et al.*, 2016). Since that publication, no further studies assessing the perception of the PC in Peru have been published.

In this context, understanding medical students’ perceptions of PC is essential, particularly in a country like Peru, where PC plays a key role in addressing public health needs and reducing inequities in access to healthcare services. Therefore, this study aims to assess medical students’ perceptions of PC and examine whether factors such as family background, social environment, and exposure to first-level care are associated with these perceptions.

## Methods

### Design and participants

A cross-sectional study was conducted with medical students from two Peruvian universities: Universidad Peruana Unión (UPeU) and Universidad Peruana Cayetano Heredia (UPCH). Data collection was carried out from July to December 2023 at UPeU (2023-II) and from April to August 2024 at UPCH (2024-I), corresponding to the second (July until December) and first (February until July) academic semesters in Peru, respectively.

Only students enrolled in the 2023-II semester (UPeU) or the 2024-I semester (UPCH), who agreed to participate, signed the informed consent form, and completed the survey questions of interest were included.

Students from the third to the seventh academic year at both universities were included. This decision was based on the fact that, beginning in the third year, students engage in practical training within primary healthcare settings, such as health centers or community-based activities, which offers them direct exposure to the context under study. This approach ensured that all participants had at least some level of experience with primary care.

### Settings

UPCH was founded in 1961 with the establishment of the Alberto Hurtado School of Medicine. Since then, it has been recognized for its academic and scientific excellence in training physicians in Peru (‘Universidad Peruana Cayetano Heredia, historia y organizacion’, 2022). This has positioned it as the leading research university in Peru, according to the Scimago Institutions Rankings (Scimago Institutions Rankings, n.d.). Between 2019 and 2020, UPCH graduated 256 physicians, and in 2024, it recorded 1421 students from the first to the seventh year of the program (‘Universidad Peruana Cayetano Heredia, historia y organizacion’, 2022).

UPeU is affiliated with the Seventh-day Adventist Church, and its human medicine program was established in 2012 with the goal of training ‘integral, missionary, and innovative physicians’. This mission aligns with the university’s principles, which are based on a biblical-Christian worldview. The first cohort, comprising approximately 25 students, graduated in 2019, and by 2024, the program reported a total of 701 medical students (Universidad Peruana Union, n.d.).

These institutions were selected for convenience, as the authors were affiliated with them, facilitating access for conducting the surveys. Furthermore, including universities with different academic profiles, one with a longstanding research focus and the other with a mission rooted in community and values-based education, offers complementary perspectives that enrich the understanding of medical students’ perceptions of primary care.

### Procedures

A self-administered survey was developed. A pilot study involving 15 medical students (5 from UPCH and 10 from UPeU) was conducted to evaluate the clarity and comprehension of the questions, ensuring that each item was interpreted correctly in terms of its intent and meaning. Based on this pilot, the questionnaire was supplemented with a definition of primary care and the services it provides, as established by Peru’s Ministry of Health (Ministerio de Salud, 2024). Necessary permissions were obtained to administer the survey at both universities.

At UPeU, a member of the research team visited classrooms during scheduled tutorial sessions in the 2023-II semester and introduced the purpose of the study to students from the third to the seventh year. Approximately 10 minutes were allocated for completing the questionnaire in person. The survey was administered directly by the research team. Participation was voluntary, informed consent was obtained prior to completion, and no personal or identifying data were collected, ensuring the anonymity of responses. The introductory section of the survey also included the contact information of the researchers affiliated with the university in case students had any questions or concerns.

At UPCH, due to the lack of time available during tutorial hours, institutional permission was obtained to disseminate the survey electronically. A member of the research team sent the survey via institutional email to students enrolled in the 2024-I semester and coordinated with class representatives to share it through their academic year group chats. The online survey included a description of the study and a consent form at the beginning. As with UPeU, no identifying data were requested, maintaining respondent anonymity, and contact information for affiliated researchers was provided for inquiries.

### Variables

The primary outcome of this study was the perception of primary care, assessed using the ‘Perception on the Labour of the First Level of Health Care’ scale (Mayta-Tristán *et al.*, 2013). This instrument consists of 11 items rated on a five-point Likert scale (ranging from 1 = strongly agree to 5 = strongly disagree), organized into three domains: (i) perceptions of physicians working in primary care; (ii) perceptions of healthcare work in primary care; and (iii) perceptions of the economic consequences of working in primary care. The scale was validated in 2013 among medical students from eight Spanish-speaking countries and demonstrated good internal consistency (*α* = 0.78) (Mayta-Tristán *et al.*, 2013). It has since been applied in studies assessing perceptions among medical students from 11 Latin American countries (Pereyra-Elías *et al.*, 2016), as well as among general practitioners in Peru during their rural service (Bendezu-Quispe *et al.*, 2021).

For the final score, the sum of all item scores was calculated, yielding a total ranging from 11 to 55 points, where a higher score indicated a more unfavorable perception. Subsequently, the total score was divided into tertiles, with the first tertile considered as a favorable perception, following a methodology previously applied in a study involving 11 countries. This approach facilitates interpretation by distinguishing between participants with the most positive views and the rest of the sample, while also allowing consistency with previous literature (Pereyra-Elías *et al.*, 2016). Additionally, sociodemographic characteristics (sex, age, academic year, marital status) and formative-employment expectations (expected income, desired workplace, intention to emigrate) were collected.

### Data analysis

The statistical analysis was performed using STATA software version 18.0 (Stata Corporation, College Station, Texas, USA) (‘Stata | StataCorp LLC’ n.d.).

In the descriptive analysis, absolute and relative frequencies were reported for categorical variables, while measures of central tendency and dispersion were presented for numerical variables.

To evaluate factors associated with a favorable perception of PC and to ensure model stability while reducing sparse data bias, only variables with at least 20 individuals in each category were included in the regression analyses (Peduzzi *et al.*, 1996; ‘Regression Modeling Strategies’, 2011). Poisson regression with robust variance was used to calculate crude prevalence ratios (PR) and adjusted prevalence ratios (aPR), along with their respective 95% confidence intervals (95% CI). Variables with a *p*-value < 0.20 in the crude association analysis with the outcome were included in the multivariable model, similar to previous exploratory studies (Misgna *et al.*, 2016; Ghassab-Abdollahi *et al.*, 2020; Arage *et al.*, 2022). following methodological recommendations suggesting that a less restrictive threshold helps to avoid excluding potential confounders (MacCallum *et al.*, 2002; ‘Regression Modeling Strategies’, 2011). A *p*-value < 0.05 was considered statistically significant in all models.

### Ethical considerations

The study was reviewed and approved by the ethics committees of UPeU (2023-CEUPeU-009) and UPCH (CIEI-066-06-24) prior to its implementation.

All participants received an informed consent form before completing the survey. The form included detailed explanations of the study’s objectives, procedures, potential risks, and possible benefits. Participants who agreed to take part provided voluntary informed consent before accessing the survey. They were assured that their responses would remain confidential and accessible only to the researchers for the purposes of this study.

## Results

A total of 456 medical students from the two universities were surveyed. Of these, 30 participants were excluded due to incomplete data, and 8 were excluded for being outside the target academic years. Thus, 418 participants were included in the final analysis. At UPeU, 314 students (77.5%) participated out of 403 enrolled from the third to seventh academic years. At UPCH, 104 students (10.6%) participated out of 981 enrolled in the same academic years (Supplementary Material 1).

Of the participants, 60.8% were women, and the average age was 23.4 years. A total of 86% reported having no relatives and 89.5% reported having no faculty members linked to PC; however, 50.9% indicated having close friends associated with PC. Regarding prior training in PC, 71.0% had attended at least one extracurricular course or seminar. Only 2.2% expressed a desire to work in primary care centers, and 92.6% intended to emigrate abroad to practice professionally. The average perception score of PC was 31.7 points **(**Table 1 **)**. Additionally, approximately 50% of the participants were found to have a favorable perception when analyzed by domains (Supplementary Material 2).

**Table 1.** Characteristics of the included medical students (*n* = 418)

Characteristic	*N* (%)
Sex	
Female	254 (60.8)
Male	164 (39.2)
Age (years)*	23.4 ± 2.5
Academic year	
Third-fourth year	147 (35.2)
Fifth-sixth year	209 (50.0)
Seventh year	62 (14.8)
University	
U Peruana Unión	314 (75.1)
U Peruana Cayetano Heredia	104 (24.9)
Place of birth	
Metropolitan Lima	215 (51.4)
Province	157 (37.6)
Foreign country	46 (11.0)
Birthplace characteristics	
Urban	405 (96.9)
Rural	13 (3.11)
Marital status	
Single	406 (97.1)
Cohabiting	12 (2.9)
Number of children	
None	409 (97.8)
One	9 (2.2)
Family member involved in PC	
No	360 (86.1)
Yes	58 (13.9)
Close friend involved in PC	
No	185 (44.3)
Yes	233 (55.7)
Professor involved in PC	
No	374 (89.5)
Yes	44 (10.53)
Courses/Seminars on PC	
No	121 (29.0)
Yes	297 (71.0)
External rotation in PC centers	
No	315 (75.4)
Yes	103 (24.6)
Health promotion community volunteer	
No	369 (88.3)
Yes	49 (11.7)
Second health-related degree	
No	405 (96.9)
Yes	13 (3.1)
Expected monthly salary (in soles)	
Less than 7000	147 (35.2)
Between 7000 and 9000	151 (36.1)
More than 9000	120 (28.7)
Expected workplace	
Hospital	303 (72.5)
Clínic	100 (23.9)
PC center	9 (2.2)
Other	6 (1.4)
Preferred area after graduation	
Medical	213 (50.9)
Surgical	193 (46.2)
Other	12 (2.9)
Willingness to emigrate for practice	
No	31 (7.4)
Yes	387 (92.6)
Perception of PC	
Unfavorable (30–55 points)	272 (65.1)
Favorable (11–29 points)	146 (34.9)

* Mean ± standard deviation, PC: Primary care.

**Figure 1.**
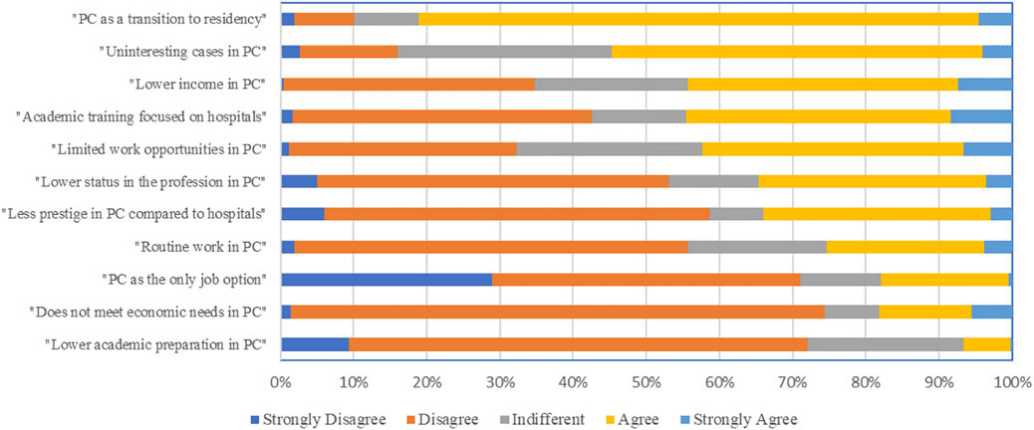
Frequency of each item included in the survey PC.

We observed that 82% of participants agreed or strongly agreed that PC was a stepping stone toward residency, 55% agreed that they encountered uninteresting cases in PC, 44% believed that those working in PC earned lower incomes and had academic training more focused on hospital work, and 35% felt that PC work had lower status compared to hospital work. Futhermore, only 6% agreed that PC professionals receive less training compared to those working in hospitals. (Figure 1 and Table 2).

**Table 2.** Scores for each question and domain of the survey (*n* = 418)

	Domains and questions	Median (IQR)	Mean ± SD
Domain 1: Perceptions of physicians working in PC, Score min-max (5–25)		12 (11–17)	13.7 ± 3.4
Domain 2: Perceptions of clinical work in PC, Score min-max (4–20)		12 (10–14)	12.3 ± 2.6
Domain 3: Perceptions of the economic consequences of working in PC, Score min-max (2–10)		5 (4–6)	5.6 ± 1.6
Total score		32 (28–34)	31.7 ± 5.8
D1	1. Do you think a doctor working in primary care has less prestige in society than a doctor who works in a hospital?	2 (2–4)	2.7 ± 1.1
D1	2. Do you think that a doctor working in primary care did not have any other options?	2 (1-3)	2.2 ± 1.1
D1	3. Do you think that a doctor working in primary care is less academically prepared than a doctor who works in a hospital?	2 (2–3)	2.3 ± 0.7
D3	4. Do you think that a doctor working in primary care has a lower income than a doctor who works in a hospital?	3 (2–4)	3.1 ± 1.0
D1	5. Do you think that a doctor working in primary care has a lower status within the medical profession, compared to a doctor who works in a hospital?	2 (2-4)	2.8 ± 1.1
D3	6. Do you think that if you worked in primary care in your country, you would not be able to meet your economic needs?	2 (2–3)	2.5 ± 0.9
D1	7. Do you think that primary care labor is a transition period between finishing medical school and the specialty (residence)?	4 (4–4)	3.7 ± 0.7
D2	8. Do you think that in primary care, physicians see uninteresting cases compared to hospital activities?	4 (3–4)	3.4 ± 0.8
D2	9. Do you think that primary care labor is a routine compared to hospital activities?	2 (2–4)	2.7 ± 0.9
D2	10. Do you think that primary care labor is limited compared to hospital activities?	3 (2–4)	3.1 ± 0.9
D2	11. Do you think that the academic training you received at medical school is oriented towards hospital-activities rather than primary care labor?	3 (2–4)	3.1 ± 1.0

D1: Domain 1, D2: Domain 2, D3: Domain 3, PC: Primary healthcare, IQR: Interquartile range, SD: Standard Deviation.

When assessing factors associated with a favorable perception of PC, we found that in the adjusted model, being enrolled at UPeU (aPR: 3.35, 95% CI: 1.85–6.05) and having completed an external rotation in PC centers (aPR: 1.36, 95% CI: 1.03–1.80) were significantly associated with a favorable perception of PC (Table 3).

**Table 3.** Associated factors with favorable perceptions of primary care labor (*n* = 418)

Characteristics	Unfavorable (*n* = 272)	Favorable (*n* = 146)	Bivariate analysis		Adjusted model*	
			PR	CI 95%	aPR	CI 95%
Sex						
Male	108 (65.8)	56 (34.2)	Ref.	–	Ref.	–
Female	164 (64.6)	90 (35.4)	1.03	(0.79–1.36)	–	–
Age, in years	23.5 ± 2.4	23.3 ± 2.7	0.97	(0.92–1.03)	–	–
Academic year						
Third-fourth year	89 (60.5)	58 (39.5)	Ref.	–	–	–
Fifth-sixth year	139 (66.5)	70 (33.5)	0.84	(0.64–1.11)	–	–
Seventh year	44 (71.0)	18 (29.0)	0.73	(0.47–1.14)	–	–
University						
U Peruana Cayetano Heredia	89 (85.6)	15 (14.4)	Ref.	–	Ref.	–
U Peruana unión	183 (5.3)	131 (41.7)	2.89	(1.77–4.70)	3.35	(1.85–6.05)
Place of birth						
Metropolitan Lima	141 (65.6)	74 (34.4)	Ref.	–	–	–
Province	105 (66.9)	52 (33.1)	0.96	(0.72–1.28)	–	–
Foreign country	26 (56.5)	20 (43.5)	1.26	(0.86–1.84)	–	–
Family member involved in PC						
No	230 (63.9)	130 (36.1)	Ref.	–	–	–
Yes	42 (72.4)	16 (27.6)	0.76	(0.49–1.18)	–	–
Close friend involved in PC						
No	116 (62,7)	69 (37,3)	Ref.	–	–	–
Yes	156 (66.9)	77 (33.1)	0.88	(0.68–1.15)	–	–
Professor involved in PC						
No	263 (63.1)	138 (36.9)	Ref.	–	Ref.	–
Yes	36 (81.8)	8 (18.2)	0.49	(0.25–0.93)	0.96	(0.48–1.91)
Courses/seminars on PC						
No	89 (73.55)	32 (26.45)	Ref.	–	Ref.	–
Yes	183 (61.6)	114 (38,4)	1.41	(1.04–2.02)	0.91	(0.63–1.30)
External rotation in PC centers						
No	211 (67.0)	104 (33.0)	Ref.	–	Ref.	–
Yes	61 (59.2)	42 (40.8)	1.23	(0.93–1.63)	1.36	(1.03–1.80)
Health promotion community volunteer						
No	235 (63.7)	134 (36.3)	Ref.	–	Ref.	–
Yes	37 (75.5)	12 (24.5)	0.67	(0.40–1.12)	1.11	(0.61–2.00)
Expected monthly salary (in soles)						
Less than 7000	92 (62.6)	55 (37.4)	Ref.	–	–	–
Between 7000 and 9000	105 (69.5)	46 (30.5)	0.81	(0.59–1.12)	–	–
More than 9000	75 (62.5)	45 (37.5)	1.00	(0.73–1.36)	–	–
Preferred area after graduation						
Medical	136 (63.8)	77 (36.2)	Ref.	–	–	–
Surgical	126 (65.3)	67 (34.7)	0.96	(0.73–1.24)	–	–
Other	10 (83.3)	2 (16.7)	0.46	(0.13–1.65)	–	–
Willingness to emigrate for practice						
Yes	255 (65.9)	132 (34.1)	Ref.	–	Ref.	–
No	17 (54.8)	14 (45.2)	1.32	(0.87–1.99)	1.14	(0.75–1.71)

PR = Prevalence Ratio; Ref. = Reference group; PC: Primary Care.

* Adjusted for university, courses, externships, volunteering, professor in PC, and intention to emigrate.

A sensitivity analysis was conducted using the variable of interest as a numerical value, based on the total survey score. A significant association was observed for being enrolled at UPeU (adjusted coefficient: −6.09, 95% CI: −8.28 to −3.89) and being female (adjusted coefficient: −1.13, 95% CI: −2.16 to −0.10). However, completing an external rotation in PC centers was not statistically significant in the final model (adjusted coefficient: 0.17, 95% CI: −1.28 to 1.63) (Supplementary Material 2).

## Discussion

On average, students scored 31.7 points on the PC perception scale used. A total of 82% agreed or strongly agreed that PC is merely a preparatory stage for residency, 55% agreed that cases encountered in PC are less interesting, and only 6% agreed that PC professionals receive less training compared to those working in hospitals. Factors associated with a favorable perception were attending a specific university and having completed an external rotation in PC centers.

### Context

In Peru, according to the National Superintendence of Higher University Education in 2021, there are 45 medical schools nationwide, with most located in the capital city (Superintendencia Nacional de Educación Superior Universitaria, n.d.). The medical degree program spans seven years, with the final year dedicated to clinical internships in hospitals.

In recent years, training in PC centers or related courses has been increasingly introduced. However, this inclusion is not uniform or mandatory across all universities. Most medical education programs remain focused on theoretical content rather than fostering comprehensive practical skills, limiting exposure to real-world primary healthcare settings during training (Bermúdez-García *et al.*, 2020).

### Comparison with previous studies

Two previous studies used the same scale as ours. One study conducted in 2016 among third- and fifth-year medical students, including 11 countries and 31 medical schools in Peru, with a total of 3768 participants, reported a median score of 33 points, similar to the 32-point median found in our study. By domain, 39.9% had a favorable perception of PC professionals, 46.1% of PC clinical work, and 47.8% of the economic implications of working in PC. In contrast, our study reported higher percentages in these domains, with 56.0%, 42.1%, and 51.0%, respectively (Pereyra-Elías *et al.*, 2016). These differences may reflect variations in the population characteristics, as we included different academic years.

The other study, conducted in 2016 among 215 physicians starting the Rural and Urban Marginal Health Service in Peru, reported an initial average score of 33.3 points, which increased to 36.8 points after eight months of work. These values are similar to the average score of 31.7 points in our study. The item with the highest score was the perception that academic training at universities is oriented more toward hospital work than PC. This was followed by the idea that PC is limited compared to hospital activities and concerns about economic income. The lowest-scoring items included the perception of lower social prestige in PC, lesser academic preparation, and viewing PC work as a last-resort job. In our study, the highest score corresponded to the perception of PC as a transitional stage toward residency, while the lowest score was related to the perception that PC work in the country does not meet economic needs and is chosen due to a lack of alternatives (Bendezu-Quispe *et al.*, 2021).

### Associated factors

We found that being affiliated with UPeU was associated with a better perception of PC. At UPeU, third-year students participate in a community externship that includes public health theory and involvement in health centers and prevention programs. However, after this course, no additional subjects directly addressing PC are included in the curriculum. At UPCH, third-year students take a basic epidemiology and community health course, which involves engagement with PC and the promotion and prevention of common diseases. In the fourth and fifth years, clinical practice-oriented courses include 3–4 weeks of variable PC involvement (ranging from 4 to 12 days). In the sixth year, one of the ten mandatory rotations is focused on primary care. Similarly, during the internship, a rotation in first-level care is included, although its duration varies depending on the institution where the student completes their internship.

In both universities, opportunities to rotate in primary care settings during the internship are also limited. Students interning at institutions such as EsSalud or private clinics often spend less time in first-level care, as their activities are mainly hospital-based. In contrast, those interning at Ministry of Health (MINSA) hospitals have longer access to primary care centers, providing them with greater exposure to primary care and community-level prevention programs (Ministerio de Salud, 2022). This finding highlights an important opportunity for universities to reevaluate and strengthen how primary care is integrated into the medical curriculum. Previous studies have observed that offering longitudinal experiences in PC (Henschen *et al.*, 2020), especially through activities embedded throughout the academic years, starting from the early stages of training can enhance clinical and patient-centered learning, improve teamwork skills, and promote shared learning experiences, all without affecting academic performance (Poncelet *et al.*, 2011; Liang *et al.*, 2021; Chan *et al.*, 2024).

We found that completing an external rotation was associated with a more favorable perception of PC. There is growing evidence supporting the benefits of incorporating practical rotations or longitudinal programs in PC into undergraduate education to enhance students’ positive perception (Pfarrwaller *et al.*, 2015; Budhathoki *et al.*, 2017; Aparicio Rodrigo *et al.*, 2021; Jungbauer *et al.*, 2021). Similarly, a 2016 study in Cuba among third- and sixth-year students evaluated their satisfaction with working in Primary Healthcare and found that education in this setting positively influenced acquired skills, with 90.7% passing regular exams (Mendoza Molina *et al.*, 2019). Another study in Spain, conducted among fifth- and sixth-year students, observed that pediatric rotations in PC positively influenced their experience and resulted in a favorable evaluation of this field (Aparicio Rodrigo *et al*., 2020).

This improvement could be attributed to external rotations providing personal patient interactions and broader learning opportunities compared to hospitals, where patients typically present with more complex conditions (Worley *et al.*, 2000; Worley *et al.*, 2006). Furthermore, these rotations may facilitate students’ integration into the healthcare system, influencing their perception while improving community-oriented knowledge and attitudes (Worley *et al.*, 2006). In addition, Universities and policymakers have the chance to make a meaningful impact by offering support such as scholarships, loan forgiveness programs, or mentorship in primary care from the early stages of training. These kinds of initiatives may give students the encouragement and reassurance they need to confidently choose a path in primary care. At the same time, incorporating more hands-on, innovative learning experiences into medical education is essential to help future physicians develop the practical skills and human perspective needed to care for people in real-world, community-based settings (Institute of Medicine (US) Committee to Study Strategies for Supporting Graduate Medical Education for Primary Care Physicians in Ambulatory Settings 1989; Butkus *et al.*, 2016).

However, the sensitivity analysis showed no statistically significant association between external rotation and PC perception. This discrepancy might be due to differences in how the outcome was defined, the continuous score’s limited ability to capture meaningful thresholds of favorable perception, or greater variability in responses across the full scale. So, while rotations seem to influence perception, these results should be interpreted with some caution.

### Limitations and strengths

This study has some limitations. First, as a cross-sectional design, it does not permit causal inferences. For instance, although we observed an association between external rotations and more favorable perceptions of primary care, the direction of this relationship remains unclear, and it is possible that students already inclined toward primary care are more likely to pursue such rotations, or that unmeasured variables influenced both factors. Nevertheless, as an exploratory study, these findings can serve as a foundation for generating hypotheses for future research.

Second, only 104 out of 981 medical students from UPCH participated in the survey, representing a relatively small proportion of the student body. As a result, caution should be exercised when generalizing these findings to the entire university population, as the sample may not fully capture the diversity of perspectives present across all academic years and campuses.

Third, all variables were self-reported, which may introduce recall bias or social desirability bias. Participants might have misremembered past experiences or responded in a manner they perceived as more acceptable or favorable, potentially affecting the accuracy of the data. This limitation is particularly relevant when assessing subjective constructs such as perceptions and attitudes.

Fourth, in the absence of a validated cut-off for our PC perception scale, we categorized scores into tertiles in line with Pereyra-Elías *et al.* (Pereyra-Elías *et al.*, 2016). While this approach enhances interpretability and preserves consistency with previous research, tertile thresholds are inherently sample-specific and may obscure finer distinctions in perception, thereby limiting direct comparability across studies. Future research should seek to validate and refine these thresholds to improve the scale’s generalizability and interpretability.

An additional limitation relates to differences in survey administration procedures. At UPeU, the survey was conducted in person, whereas at UPCH, it was distributed online. In-person administration may have introduced social desirability bias, while online distribution, although potentially offering greater privacy, may have affected participation rates. Moreover, we acknowledge that variations in curricula (particularly in the timing, content, and intensity of primary care exposure) across academic years or between the two universities could have influenced students’ perceptions.

Despite these limitations, our study stands out as one of the few conducted in Peru and in Latin America, emphasizing its importance in a regional context where research on this topic is limited, especially regarding its implications for the medical workforce in community settings.

### Conclusions

In conclusion, a higher percentage of participants had an unfavorable perception of PC, which persisted across dimensions evaluating the physician’s role, healthcare delivery, and economic consequences. A small percentage expressed interest in working in primary care centers. Additionally, we found an association between external rotations in PC and being enrolled in a specific university with a more favorable perception. We recommend that policymakers and universities prove structured interventions (such as curricular reforms, primary care–focused internships, community-based field experiences, or longitudinal clinical rotations) to strengthen students’ understanding of the importance and potential of primary care. These strategies may help improve medical students’ perceptions and foster greater interest in primary care careers. Future research should explore the undergraduate curriculum and assess educational interventions aimed at enhancing students’ perceptions and preparedness for work in this field.

## Supporting information

Medina-Ramirez et al. supplementary material Medina-Ramirez et al. supplementary material

## Supplementary material

The supplementary material for this article can be found at https://doi.org/10.1017/S146342362610084X.
