# Supplementary material for: Perceptions of primary care among medical students in Lima, Peru: a cross-sectional study in two universities
Source: Prim Health Care Res Dev. 2026 Mar 9;27:e36. doi: 10.1017/S146342362610084X (PMC13080538; doi:10.1017/S146342362610084X)
Supplement: Medina-Ramirez et al. supplementary material [file S146342362610084Xsup001.docx]

**Supplementary Material**

Supplementary Material 1. Sampling Frame and Sample Size of the Two Universities Included

Supplementary Material 2. Perceptions by Domains Regarding the PC (n=418)

Supplementary Material 3. Factors Associated with Survey Scores on Perception of Primary Care Labor (n=418)

| Supplementary Material 1. Sampling Frame and Sample Size of the Two Universities Included | | |
| --- | --- | --- |
| Academic year | UPEU: 2023-II  Surveyed students / enrolled students | UPCH: 2024-I  Surveyed students / enrolled students |
| 3rd year | 47//80 = 58.7% | 17/ 250 = 6.8% |
| 4th year | 54/80 = 67.5% | 29/ 225 = 12.8% |
| 5th year | 77/90 = 85.5% | 40/ 145 = 27.6% |
| 6th year | 78/90 = 86.6% | 14/ 172 = 8.1% |
| 7th year | 58/63 = 92.1% | 4/ 189 = 2.1% |
| Total | 314/403 = 77.5% | 104/981= 10.6% |

| Supplementary Material 2. Perceptions by Domains Regarding the PC (n=418) | | |
| --- | --- | --- |
| Domains | | N (%) |
| Perceptions about the PC physician | |  |
|  | Unfavorable (13-25 points) | 184 (44,0) |
|  | Favorable (5-12 points) | 234 (56,0) |
|  |  |  |
| Perceptions about PC labor itself | |  |
|  | Unfavorable (12-20 points) | 242 (57,9) |
|  | Favorable (4-11 points) | 176 (42,1) |
|  |  |  |
| Perceptions about economic consequences of PC labor | |  |
|  | Unfavorable (6-10 points) | 205 (49,0) |
|  | Favorable (2-5 points) | 213 (51,0) |
| PC: Primary Care | | |

| Supplementary Material 3. Associated factors with Survey Scores on Perception of Primary Care Labor (n=418) | | | | |
| --- | --- | --- | --- | --- |
| Characteristic | | Mean ± Standard Deviation | Crude Coefficient (95% CI) | Adjusted Coefficient (95% CI) |
| Sex | |  |  |  |
|  | Male | 32.5 ± 6.7 | Ref. | Ref. |
|  | Female | 31.1 ± 5.1 | -1.42 (-2.63 a -0.20) | **-1.13 (-2.16 a -0.10)** |
| Age, in years | | 23.4 ± 2.5 | -0.09 (-0.33 a 0.15) | - |
| Academic year | |  |  |  |
|  | Third-fourth year | 31.4 ± 6.4 | Ref. | - |
|  | Fifth-sixth year | 31.8 ± 5.2 | 0.42 (-0.84 a 1.60) | - |
|  | Seventh year | 32.1 ± 6.4 | 0.69 (-1.22 a 2.60) | - |
| University | |  |  |  |
|  | U Peruana Cayetano Heredia | 36.5 ± 6.7 | Ref. | Ref. |
|  | U Peruana Unión | 30.1 ± 4.5 | -6.44 (-7.83 a -5.06) | **-6.09 (-8.28 a -3.89)** |
| Place of birth | |  |  |  |
|  | Metropolitan Lima | 32.2 ± 6.1 | Ref. | Ref. |
|  | Province | 31.4 ± 5.6 | -0.77 (-1.98 a 0.43) | -0.16 (-1.72 a 0.90) |
|  | Foreign country | 30.5 ± 4.9 | -1.60 (-3.25 a 0.39) | 0.03 (-1.55 a 1.61) |
| Family member involved in PHC | | |  |  |
|  | No | 31.2 ± 5.6 | Ref. | Ref. |
|  | Yes | 34.3 ± 6.5 | 3.02 (1.24 a 4.81) | 0.41 (-.1.36 a 2.20) |
| Close friend involved in PHC | | |  |  |
|  | No | 32.2 ± 7.1 | Ref. | Ref. |
|  | Yes | 31.3 ± 4.6 | -0.90 (-2.08 a 0.28) | 0.69 (-0.50 a 1.89) |
| Professor involved in PHC | | |  |  |
|  | No | 31.3 ± 5.5 | Ref. | Ref. |
|  | Yes | 35.3 ± 6.9 | 4.09 (1.97 a 6.21) | 0.18 (-2.47 a 2.83) |
| Courses/Seminars on PHC | | |  |  |
|  | No | 34.3 ± 7.2 | Ref. | Ref. |
|  | Yes | 30.6 ± 4.8 | -3.72 (-5.12 a -2.32) | -0.21 (-2.12 a 1.69) |
| External rotation in PHC centers | | |  |  |
|  | No | 31.6 ± 5.5 | Ref. | - |
|  | Yes | 31.8 ± 6.8 | 0.17 (-1.28 a 1.63) | - |
| Health promotion community volunteer | | |  |  |
|  | No | 31.2 ± 5.3 | Ref. | Ref. |
|  | Yes | 35.1 ± 8.1 | 3.89 (1.56 a 6.22) | -0.08 (-3.18 a 3.00) |
| Expected monthly salary (in soles) | | |  |  |
|  | Less than 7000 | 31.9 ± 6.4 | Ref. | - |
|  | Between 7000 and 9000 | 31.4 ± 4.7 | -0.55 (-1.84 - 0.74) | - |
|  | More than 9000 | 31.8 ± 6.3 | -0.07 (-1.61 - 1.46) | - |
| Preferred area after graduation | | |  |  |
|  | Medical | 31.7 ± 6.0 | Ref. | Ref. |
|  | Surgical | 31.3 ± 5.2 | -0.42 (-1.52 a 0.66) | -0.31 (-1.32 a 0.69) |
|  | Other | 37.7 ± 9.0 | 6.02 (1.02 a 11.01) | 2.50 (-1.20 a 8.22) |
| Willingness to emigrate for practice | | | |  |
|  | Yes | 31.8 ± 5.8 | Ref. | Ref. |
|  | No | 29.8 ± 5.2 | -2.01 (-3.09 a -0.09) | -1.78 (-3.58 a 0.02) |
| Ref. = reference group. | | | |  |
| *Adjusted for sex, university, courses, family member, friend, and professor working in PHC, volunteering, preferred area after graduation, and willingness to migrate for professional practice | | | | |
| PC: Primary Care | | | |  |
